# The Association Between Lentiform Nucleus Function and Cognitive Impairments in Schizophrenia

**DOI:** 10.3389/fnhum.2021.777043

**Published:** 2021-10-21

**Authors:** Ping Li, Shu-Wan Zhao, Xu-Sha Wu, Ya-Juan Zhang, Lei Song, Lin Wu, Xiao-Fan Liu, Yu-Fei Fu, Di Wu, Wen-Jun Wu, Ya-Hong Zhang, Hong Yin, Long-Biao Cui, Fan Guo

**Affiliations:** ^1^Medical Imaging Department 1, Xi’an Mental Health Center, Xi’an, China; ^2^Department of Radiology, Xijing Hospital, The Fourth Military Medical University, Xi’an, China; ^3^Department of Clinical Psychology, School of Medical Psychology, The Fourth Military Medical University, Xi’an, China; ^4^Department of Psychiatry, Xijing Hospital, The Fourth Military Medical University, Xi’an, China; ^5^Department of Radiology, The Second Medical Center, Chinese PLA General Hospital, Beijing, China

**Keywords:** schizophrenia, lentiform nucleus, cognition, magnetic resonance imaging, phenotype

## Abstract

**Introduction:** Cognitive decline is the core schizophrenia symptom, which is now well accepted. Holding a role in various aspects of cognition, lentiform nucleus (putamen and globus pallidus) dysfunction contributes to the psychopathology of this disease. However, the effects of lentiform nucleus function on cognitive impairments in schizophrenia are yet to be investigated.

**Objectives:** We aim to detect the fractional amplitude of low-frequency fluctuation (fALFF) alterations in patients with schizophrenia, and examine how their behavior correlates in relation to the cognitive impairments of the patients.

**Methods:** All participants underwent magnetic resonance imaging (MRI) and cognitive assessment (digit span and digit symbol coding tests). Screening of brain regions with significant changes in fALFF values was based on analysis of the whole brain. The data were analyzed between Jun 2020 and Mar 2021. There were no interventions beyond the routine therapy determined by their clinicians on the basis of standard clinical practice.

**Results:** There were 136 patients (75 men and 61 women, 24.1 ± 7.4 years old) and 146 healthy controls (82 men and 64 women, 24.2 ± 5.2 years old) involved in the experiments seriatim. Patients with schizophrenia exhibited decreased raw scores in cognitive tests (*p* < 0.001) and increased fALFF in the bilateral lentiform nuclei (left: 67 voxels; x = −24, y = −6, z = 3; peak *t*-value = 6.90; right: 16 voxels; x = 18, y = 0, z = 3; peak *t*-value = 6.36). The fALFF values in the bilateral lentiform nuclei were positively correlated with digit span-backward test scores (left: *r* = 0.193, *p* = 0.027; right: *r* = 0.190, *p* = 0.030), and the right lentiform nucleus was positively correlated with digit symbol coding scores (*r* = 0.209, *p* = 0.016).

**Conclusion:** This study demonstrates that cognitive impairments in schizophrenia are associated with lentiform nucleus function as revealed by MRI, involving working memory and processing speed.

## Introduction

Schizophrenia is a chronic mental illness affecting more than 20 million people all over the world (James et al., 2018). In schizophrenia, cognitive decline is a phenomenon related to the disorder. Schizophrenia has been considered to be a cognitive illness (Kahn and Keefe, 2013), in which cognitive decline is the core symptom (Kahn, 2019). Most recent longitudinal studies have shown 10- and 20-year progressive decline in cognitive functioning in patients with schizophrenia and other psychotic disorders (Zanelli et al., 2019; Fett et al., 2020). Cognitive function is impaired across almost all domains (Kern et al., 2011; Georgiades et al., 2017; Zhang et al., 2019) and contributes substantially to the long-term outcome associated with schizophrenia (Lepage et al., 2014; Mucci et al., 2021), highlighting cognitive symptoms as important targets for treatment.

Previous studies have demonstrated that patients with first-episode schizophrenia showed cognitive deficits across all cognitive domains, particularly in processing speed (Kern et al., 2011; Georgiades et al., 2017; Zhang et al., 2019). Working memory is the impaired cognitive domain that enters most frequently in the second position (Kern et al., 2011; Georgiades et al., 2017), and it is a neurocognitive impairment that differs between first-episode and chronic schizophrenia (McCleery et al., 2014). Taken together, we selected digit span (working memory) and digit symbol coding tests (processing speed) in this study. Taking the dysconnection hypothesis (Friston et al., 2016) and the therapeutic value of neuromodulation (Guan et al., 2020; Xiu et al., 2020) into consideration, neuroimaging study is urgently needed. However, the underlying brain structural and functional mechanisms for the cognitive symptoms remain to be identified (McCutcheon et al., 2020).

As a part of the basal ganglia, the lentiform nucleus (LN) is a lens-shaped, bilateral structure in the basal ganglia bounded by the internal and external capsules and has three components: the internal and external globus pallidus and the putamen (Hibar et al., 2013). The LN is implicated in several degenerative and psychiatric disorders (Obeso et al., 2000; Ellison-Wright et al., 2008). The housing of dopaminergic neurons in the LN explains its involvement in the neuropathology of schizophrenia, as a dopaminergic disorder (Brisch et al., 2014). Basal ganglia dysfunction has been suggested to be involved in the cognitive impairments of schizophrenia, as the dysfunction of cortical, striatal, and thalamocortical dopamine signaling circuits could lead to cognitive deficits (Simpson et al., 2010; Krabbe et al., 2015). Although the studies have suggested that the LN might be functionally linked to cognitive function in schizophrenia including attention, working memory, reward, and executive functions (Vatansever et al., 2016), the direct evidence remains unclear and has yet to be determined, especially in *in vivo* study with patients.

Magnetic resonance imaging (MRI) techniques provide promising tools to allow for exploring neural underpinnings behind this disease. Among these studies, the basal ganglia attracted particular attention, as it seems to be associated not only with the clinical manifestation of the disease but also cognitive information processing (Delvecchio et al., 2018). Previous studies prefer to focus on the structural changes of the LN area, but a few studies have shown a significant correlation between subcortical regions of interest in function (Luo et al., 2018; Fan et al., 2019; Tikasz et al., 2019). Hartberg et al. (2011) have proved that there is a negative correlation between putamen volume and verbal memory in patients with schizophrenia. Previous studies have been focused on either the functional striatal abnormalities instead of LN function in schizophrenia patients or exploring the correlation between striatal structural changes and working memory function. Further studies are needed to show the direct relationship between LN and cognitive function in schizophrenia patients.

Both the amplitude of low frequency fluctuations (ALFF) and the fraction amplitude of low frequency fluctuations (fALFF) can reflect the intensity of spontaneous activity in brain areas. The fALFF is a modified index of the ALFF, being less likely to produce any noise and more sensitive and specific to the detection of spontaneous brain activities in comparison to the ALFF (Chai et al., 2020). Many studies have found that the fALFF is associated with cognitive symptoms of schizophrenia (Fryer et al., 2015; Sui et al., 2015). A recent study, aimed to identify multimodal biomarkers for quantifying and predicting cognitive performance in individuals with schizophrenia and healthy controls, has found that fALFF features were more sensitive to cognitive domain differences (Sui et al., 2018).

Therefore, we aim to detect fALFF alterations in patients with schizophrenia. Specifically, given the involvement of the LN in the pathophysiology of schizophrenia, abnormal functioning of the LN can be strongly associated with the development of cognitive impairment. In the current study, we used the fALFF to determine the relevance of abnormal LN function on cognitive impairments in schizophrenia.

## Materials and Methods

### Participants

This study was approved by the Institutional Ethics Committee, First Affiliated Hospital (Xijing Hospital) of the Fourth Military Medical University. Each participant gave written informed consent after receiving a complete description of this study. Between April 8, 2015 and June 18, 2020, 141 patients with schizophrenia were recruited from the Department of Psychiatry at Xijing Hospital. There were also 146 matched healthy controls, who were enrolled through advertising. The participants were diagnosed on the basis of the Diagnostic and Statistical Manual of Mental Disorders, Fifth Edition (DSM-5), and consensus diagnoses were made by two experienced clinical psychiatrists using all the available information. At the time of scanning, all the subjects underwent the Positive and Negative Syndrome Scale (PANSS) and cognitive assessment (digit span and digit symbol coding test). They were all right-handed, and their biological parents were of the Han Chinese ethnic group. The exclusion criteria for patients were as follows: (1) the presence of another psychiatric disorder; (2) a history of repetitive transcranial magnetic stimulation, transcranial current stimulation, or behavioral treatment; (3) a history of clinically significant neurological, neurosurgical, or medical illnesses; (4) substance abuse within the prior 30 days or substance dependence within the prior 6 months; (5) pregnancy or any other MRI contraindications, e.g., cardiac pacemakers and other metallic implants; (6) unwillingness to undertake the scanning. Exclusion criteria for healthy controls were as follows: (1) the presence of any psychotic syndrome; (2) a history of receiving antipsychotics, repetitive transcranial magnetic stimulation, transcranial current stimulation, or behavioral treatment; the remaining (3), (4), (5), and (6) were the same as the exclusion criteria for patients.

### Cognitive Assessment

We used the Wechsler Adult Intelligence Scale revised in China (WAIS-RC) to assess cognition by digit span (forward and backward) and digit symbol coding tests. For the forward digit span task, the subject was initially required to repeat a string of numbers after the researcher read them out. If the subject is able to repeat the string correctly, then they would be asked to proceed to the next string, which would have its length increased by one; if not, a second test would be conducted with a different string of digits of the same length. If the subject is correct, the test continues with a longer string, otherwise, the test stops and the length of the string is recorded. With the backward digit span test, the subjects were asked to repeat from the last number to the first after hearing a string of numbers, and the rest of the process was consistent with the forward test. In the digit symbol coding test, the subject is required to define 10 different symbols for 10 numbers from 0 to 9. The subject is asked to write the corresponding symbols under disordered numbers within 90 s, and the number of characters written correctly is recorded. Ultimately, digit symbol coding and digit span-forward data were available for 132 patients and 56 healthy controls. In addition, one patient rejected the digit span-backward test.

### Image Acquisition

A General Electric (GE) Discovery MR750 3.0 T scanner was used to acquire images at the Department of Radiology at Xijing Hospital with a standard 8-channel head coil. A T1-weighted anatomical imaging (TR = 8.2 ms, TE = 3.2 ms, slice thickness = 1.0 mm, field of view [FOV] = 256 mm × 256 mm, matrix = 256 × 256, and flip angle = 12°) and resting-state functional MRI (TR = 2,000 ms, TE = 30 ms, slice thickness = 3.5 mm, FOV = 240 mm × 240 mm, matrix = 64 × 64, and flip angle = 90°) were performed. Further details about image acquisition are detailed in previous articles (Cui et al., 2019b; Liu et al., 2019). Participants were instructed to relax with their eyes closed but keep from falling asleep during their MRI scan.

### Data Processing

The data processing was performed using the Data Processing Assistant for Resting-State fMRI Advanced Edition (DPARSFA) V4.4^1^ with the previously published protocols (Cui et al., 2019a). First, the first 10 time points were discarded to ensure the stability of the magnetic field. Second, slice timing correction and realignment (subjects with maximum motion > 2 mm or 2° were excluded) were performed. Five patients were excluded from the study because of excessive head motion, resulting in 136 patients who were included in the following analysis. Third, the nuisance covariates that included six head motion parameters, cerebrospinal fluid signals, white matter signals, and global mean signals were regressed from the data as corrected values. Fourth, T1-weighted images were coregistered to the realigned functional images. Fifth, the coregistered images were normalized to Montreal Neurological Institute space and resampled to 3 mm × 3 mm × 3 mm voxels. Sixth, the volumes were smoothed with a Gaussian kernel (8 mm full-width half-maximum, FWHM).

### Fractional Amplitude of Low Frequency Fluctuations Analysis

We used the ALFF for directly observing local field spontaneous neural activity (Logothetis et al., 2001). The ALFF values of the subjects were calculated using the DPARSFA V4.4 (see text footnote 1). The ALFF calculation was performed using previously published protocols (Cui et al., 2019a). Finally, a filtering band-pass (0.01–0.08 Hz) was performed after calculating the ALFF. To overcome the limits of the ALFF approach, a ratio of the power of each frequency at a low-frequency range to that of the entire frequency range, known as fALFF, was obtained for the following statistical analysis (Yang et al., 2018).

### Statistical Analysis

For voxel-based comparison of the fALFF, a two-sample *t*-test in SPM12 software^2^ was used to test the statistical significance between patients and controls. A *p* < 0.05 (FWE correction) with a cluster size of more than 15 was considered as the statistical significance for the fALFF analysis. The comparison of demographical data and correlation analyses were performed in the Statistical Product and Service Solutions (SPSS, version 22.0). Demographical characteristics (age, gender, and education) and Jenkinson’s mean frame-wise displacement were regarded as covariates. A region of interest was created using the significant clusters of group comparison to extract the fALFF values. We used Pearson correlation coefficients to assess the clinical relevance between the fALFF value of the LN (putamen and globus pallidus) and cognitive capacity in patients (significance was set at *p* < 0.05).

## Results

### Demographical and Clinical Characteristics

Table 1 presents the demographic and clinical characteristics of the participants. Apart from the level of education, there was no statistically significant difference in other characteristics between patients and healthy controls.

**TABLE 1:** Demographical and clinical characteristics.

	Schizophrenia patients (*n* = 136)	Healthy controls (*n* = 146)	*p*-values
Age, y^a^	24.1 (7.4)	24.2 (5.2)	0.922
Gender, M/F^b^	75/61	82/64	0.864
Education, y^a^	12 (3)	15 (3)	<0.001
Status, FE/NFE	101/35	/	
Medication, U/T	27/109	/	
Illness duration, mon	14.7 (22.7)	/	
PANSS score		/	
Positive	21.7 (5.3)	/	
Negative	20.2 (7.3)	/	
General	43.7 (8.3)	/	
Total	85.6 (14.3)	/	

*Data are shown in mean (standard deviation). FE, first episode; NFE, non-first episode; U, untreated; T, treated. ^a^Two-sample t-test. ^b^Pearson Chi-Square test.*

### Cognitive Impairments

**FIGURE 1:**
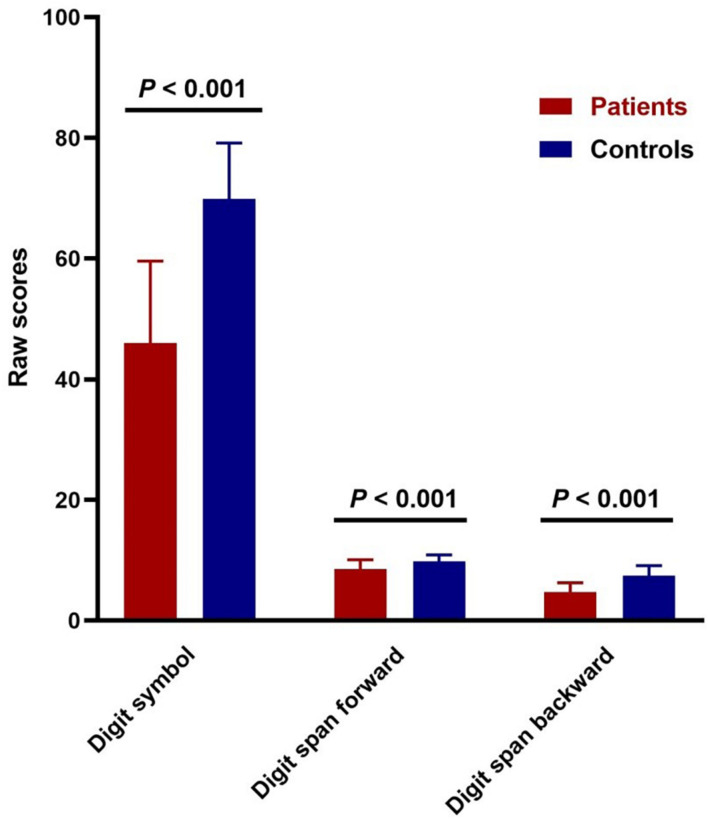
Cognitive impairments in patients with schizophrenia. Comparison of digit symbol coding test (*t* = –13.985, *p* = 3.503E-29) and digit span raw scores (forward: *t* = –5.745, *p* = 4.7247E-8; backward: *t* = –10.225, *p* = 9.7559E-20) between patients and controls.

The digit symbol coding and digit span-forward data for 132 patients and 56 controls were available. As for digit span-backward, there was one patient who refused to take the test. Two-sample *t*-testing showed significant differences in the cognitive tests (digit symbol coding, digit span-forward, and digit span-backward) between the two groups (*p* < 0.001; Figure 1).

### Disrupted Fractional Amplitude of Low-Frequency Fluctuation

**FIGURE 2:**
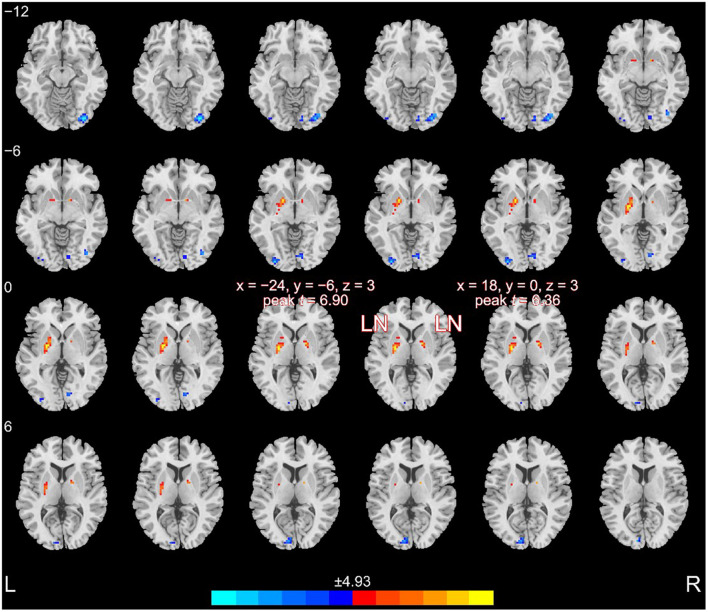
Altered fALFF in patients compared to healthy controls. Patients with schizophrenia exhibited increased fALFF in the bilateral LN.

In the whole-brain analysis, the regions with altered fALFF values are shown in Figure 2. Briefly, schizophrenia patients exhibited increased fALFF in the left LN (67 voxels; x = −24, y = −6, z = 3; peak *t*-value = 6.90) and the right LN (16 voxels; x = 18, y = 0, z = 3; peak *t*-value = 6.36). The brain regions with decreased fALFF values included the right anterior occipital gyrus (36 voxels; x = 33, y = −84, z = −12; peak *t*-value = 6.42), left middle occipital gyrus (17 voxels; x = −30, y = −90, z = −3; peak *t*-value = 5.93), left superior occipital gyrus (16 voxels; x = −9, y = −90, z = 9; peak *t*-value = 5.83), and right lingual gyrus (20 voxels; x = 12, y = −84, z = −9; peak *t*-value = 5.55).

### Correlation Between Fractional Amplitude of Low-Frequency Fluctuation and Cognitive Function

**FIGURE 3:**
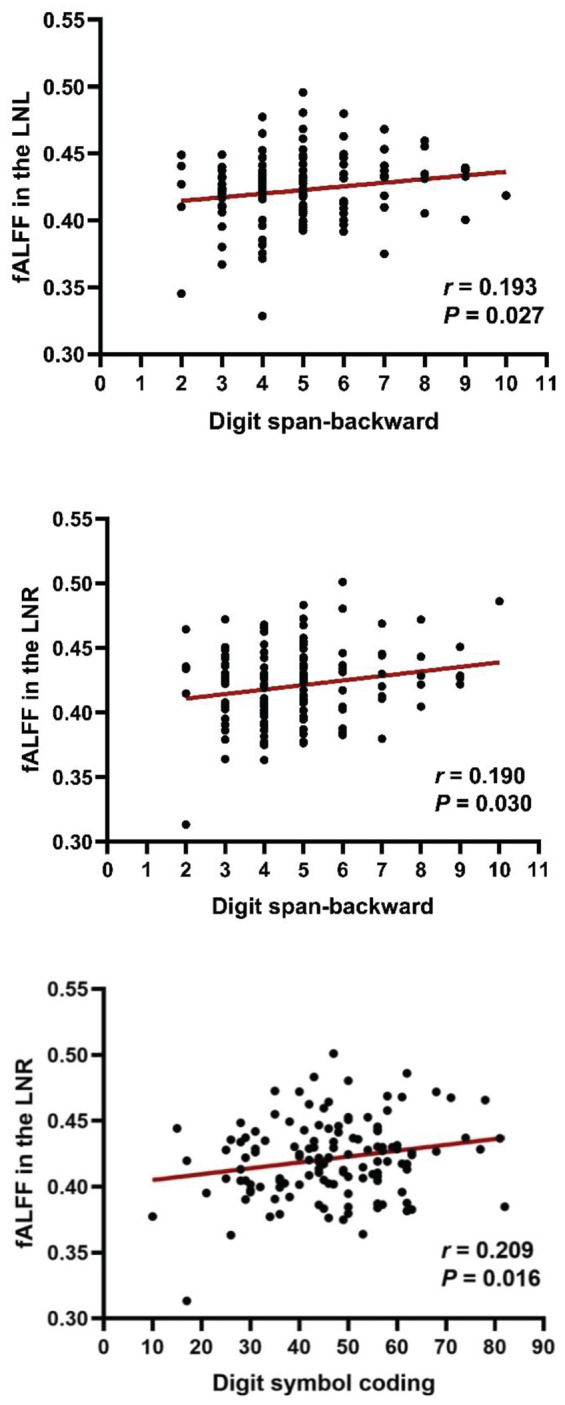
Correlation between fALFF and cognitive impairments in patients with schizophrenia. Digit span-backward test and digit symbol coding were positively correlated with fALFF values in the LN. LNL, left LN; LNR, right LN.

The fALFF values were extracted according to the mask of the bilateral LN (significant clusters of group comparison). We calculated the correlation between the cognitive scores and fALFF values in the LN of the patients and reported the (uncorrected) *p*-values because our hypothesis directly concerned these two selected regions of interest (Figure 3), as previously performed (Li et al., 2017). Considering that the scaled score tends to decrease the diversity of data, we used the raw score to present the subtle discrepancies among subjects (Xie et al., 2021). Correlation analysis showed that the digit span-backward test was positively correlated with the fALFF values (the left LN: *r* = 0.193, *p* = 0.027; the right LN: *r* = 0.190, *p* = 0.030). In addition, a positive correlation between the right LN and digit symbol coding was also demonstrated (*r* = 0.209, *p* = 0.016). However, when assessing the correlation between the digit span-forward test and the fALFF values of the LN, there was no significant association.

## Discussion

In the current study, we observed that patients and healthy controls had statistically significant differences in cognitive function. Schizophrenia patients exhibited increased fALFF in the bilateral LN. On the contrary, the brain regions with decreased fALFF included the bilateral occipital gyrus and the right lingual gyrus. Furthermore, the digit span-backward test was positively correlated with the fALFF values of the bilateral LN, and the fALFF values of the right LN were positively correlated with the digit symbol coding test.

Previous studies have shown that cognitive function is decreased in schizophrenia patients, including memory impairment, deficits in attention, and general cognition (Koshiyama et al., 2018). Although there are several neuropsychological tests for measuring processing speed impairment, which is the largest single deficit in cognitive function in schizophrenia, a meta-analysis has demonstrated that a digit symbol coding task is the most sensitive test to apply to patients with schizophrenia (Dickinson et al., 2007), and that it reflects processing speed. Moreover, digit span (forward and backward) could reflect the function of cognition especially with working memory (Conklin et al., 2000). It has been reported that memory impairment is a severe cognitive dysfunction in schizophrenia (Toulopoulou et al., 2003). Our study is consistent with previous studies showing the dysfunctional changes in cognitive function, especially in the memory of schizophrenia patients (Chen et al., 2016).

Among the different brain structures, the basal ganglia, as the subcortical nuclei rich in dopaminergic neurons, is an important structure for the neuropathology of schizophrenia, as a well-established dopaminergic disorder (Brisch et al., 2014). The basal ganglia are composed of the caudate nucleus, the LN (putamen and the globus pallidus), and the substantia nigra. The LN, as part of the basal ganglia, is not only important for the motor system but also plays a role in cognitive functions, including working memory, executive function, reward, and learning (Schroll et al., 2015). Dopamine is known to play a major part in regulating a number of cognitive functions that are impaired in schizophrenia, and research should now shift focus toward a better understanding of the role of specific striatal pathways in cognition (Conn et al., 2020; Martel and Gatti McArthur, 2020). A recent study concluded that striatal dysfunction contributes to cognitive difficulties in schizophrenia, which is supported by previous histological and neuroimaging evidence (Avram et al., 2019). Many previous studies have focused on the structural changes of LN in schizophrenia (Luo et al., 2018; Takahashi et al., 2020). Hashimoto et al. (2009) showed that schizophrenia patients had significantly lower fractional anisotropy values in the bilateral globus pallidus and left thalamus compared to controls, suggesting that schizophrenics might have microstructural abnormalities in the globus pallidus and thalamus. However, the functional role of the LN and how it affects cognitive function remains largely unknown. Several similar studies have been focused on the basal ganglia but not on the LN. And some studies have focused on the symptoms assessed by PANSS but not on cognitive function. Therefore, the direct evidence remains unclear whether the LN is linked to certain cognitive functions and has yet to be determined, especially in *in vivo* studies with patients. The purpose of the present study was to provide this evidence.

One of the main results of our study showed increased fALFF in the bilateral LN. Gong et al. (2020) also showed increased ALFF in the LN in schizophrenia patients compared with healthy controls. Another previous study found that the ALFF increased in the parietal lobule and was correlated with decreased social cognition (Sui et al., 2015). Moreover, our result showed decreased fALFF in the bilateral occipital gyrus, which is consistent with a previous study, showing that the decreased fALFF were mainly in the posterior parietal cortex and occipital cortex (Xu et al., 2015). In addition, schizophrenia patients had greater thalamic connectivity with the occipital gyrus (Ferri et al., 2018). Furthermore, abnormal morphology of the occipital gyrus may be a marker of psychiatric illness (Maller et al., 2017). A pronounced decline in gray matter volume was observed in the bilateral occipital lobe in genetic high-risk individuals and first-episode schizophrenia patients (Zhao et al., 2018). Therefore, the regional functional changes of certain brain areas, especially in the LN, might be important brain neuroimaging markers for schizophrenia patients.

Another major result of our study was the correlation between the bilateral LN and the digit span-backward test. The LN, as part of the cortico-striato-thalamocortical circuits, is important for cognitive functions, especially the attention, working memory, reward, and executive functions (Rabinovici et al., 2015; Vatansever et al., 2016). As mentioned above, the digit span backward test could reflect the function of working memory. Our result indicated the LN is correlated with working memory. In previous schizophrenia studies, there have been reports of negative performance-related functional connectivity between the left putamen and the right ventrolateral prefrontal cortices (Quide et al., 2013). Our previous study also proved the importance of putamen in positive symptoms (Cui et al., 2016, 2017), and in disease identification and the treatment response prediction of schizophrenia (Cui et al., 2018, 2020). Thus, our results add to the importance of the LN in schizophrenia, especially in cognitive function. Of note, our current results did not show a significant correlation between the digit span-forward test and the fALFF values of the LN. Our observation of the results may reflect the unique disease-related abnormalities of the LN.

Our study reflects some limitations. First, a previous study has demonstrated cognitive impairment in schizophrenia as a mediator to influence the association between negative symptoms and hippocampal morphometry (Duan et al., 2021). However, our study only focused on the relationship between the fALFF of the LN and cognitive impairments, and we were not able to answer how the LN contributes to cognitive impairments in schizophrenia. Further MRI-guidance and navigation studies combined with neuromodulation will be needed for answering this question (Wu et al., 2021). Second, antipsychotic treatments modify abnormal cerebral function in schizophrenia (Duan et al., 2020), but the effects of medication and whether the other cognitive function was affected were not investigated in the current study.

## Conclusion

In conclusion, the present investigation found the association between increased fALFF values in the bilateral LN and cognitive performance in schizophrenia patients. These findings may contribute to our understanding of the LN in schizophrenia and shed light on the development of psychological strategies to improve cognitive function *via* the new target.

## Data Availability Statement

The original contributions presented in the study are included in the article/supplementary material, further inquiries can be directed to the corresponding author/s.

## Ethics Statement

The studies involving human participants were reviewed and approved by the Medical Ethics Committee of the First Affiliated Hospital of the Fourth Military Medical University. The patients/participants provided their written informed consent to participate in this study.

## Author Contributions

FG and L-BC conceptualized the manuscript. PL, FG, and L-BC designed the study, wrote the first draft of the manuscript, and conducted the statistical analyses. S-WZ, X-SW, Y-JZ, LS, LW, X-FL, Y-FF, DW, W-JW, and Y-HZ collected and organized the primary data. L-BC and FG provided supervision in the implementation of the study. All authors provided feedback and revised the manuscript.

## Conflict of Interest

The authors declare that the research was conducted in the absence of any commercial or financial relationships that could be construed as a potential conflict of interest.

## Publisher’s Note

All claims expressed in this article are solely those of the authors and do not necessarily represent those of their affiliated organizations, or those of the publisher, the editors and the reviewers. Any product that may be evaluated in this article, or claim that may be made by its manufacturer, is not guaranteed or endorsed by the publisher.

## Footnotes

1
http://rfmri.org/DPARSF

2
http://www.fil.ion.ucl.ac.uk/spm/software/spm12/
